# Nurses’ Well-Being, Health-Promoting Lifestyle and Work Environment Satisfaction Correlation: A Psychometric Study for Development of Nursing Health and Job Satisfaction Model and Scale

**DOI:** 10.3390/ijerph17103582

**Published:** 2020-05-20

**Authors:** Hui-Chun Chung, Yueh-Chih Chen, Shu-Chuan Chang, Wen-Lin Hsu, Tsung-Cheng Hsieh

**Affiliations:** 1 Department of Nursing, Hualien Tzu Chi Hospital, Hualien 970, Taiwan; a0970332633@yahoo.com.tw; 2 Department of Nursing, Tzu Chi University, Hualien 970, Taiwan; scchang@mail.tcu.edu.tw; 3 Department of Nursing, National Taiwan University, Taipei 100, Taiwan; ychichen@ntu.edu.tw; 4 Nursing Committee, Buddhist Tzu Chi Medical Foundation, Hualien 970, Taiwan; 5 Department of Radiation Oncology, Hualien Tzu Chi Hospital, Hualien 970, Taiwan; hwl@tzuchi.com.tw; 6 Medical Department, Tzu Chi University, Hualien 970, Taiwan; 7 Institute of Medical Sciences, Tzu Chi University, Hualien 970, Taiwan

**Keywords:** turnover, health-promoting, well-being, work environment satisfaction, structural equation modeling

## Abstract

Although promoting healthy work environments to enhance staff members’ health and well-being is a growing trend, no empirical studies on such a model have been conducted in the nursing management field. The purpose of this study was to develop and validate measurement scales and a conceptual model of nurses’ well-being, health-promoting lifestyle, and work environment satisfaction (WHS). A cross-sectional survey was conducted to develop a WHS model and Nursing Health and Job Satisfaction (NHJS) scale. A total of 672 questionnaires were obtained from registered nurses by stratified random sampling for validation analysis. The percentage of total variance explained greater than 92.6%, suggesting a good ability of the scales to explain the variability in participants’ responses. The hypotheses of positive correlations among nurses’ health-promoting lifestyle, well-being, and work environment satisfaction were supported. The WHS model demonstrates the positive correlation with correlation coefficients of 0.57–0.86 among nurses’ health-promoting lifestyle, well-being, and work environment satisfaction. Nurses’ attitudes play a key role in promoting a healthy lifestyle. The most important work environment satisfaction variable for improved sense of well-being is respect from other medical staff. The findings can serve as an instrument for hospital nursing administrators to accurately assess and enhance nurses’ retention rate and health.

## 1. Introduction

Retention of nurses is a critical factor for nursing administrators, as nursing workforce is associated with patient safety and health care quality. Unfortunately, nurse turnover is a constant concern, and work environments have been revealed as the main factors causing nurse attrition and influencing nurses’ job satisfaction [1,2,3,4]. Nursing demands high dedication toward their work, however receiving insufficient positive feedback engenders physical and mental fatigue, job stress, workplace fatigue, health problems, and family discord. These factors are related to the high attrition rate amongst nurses [5].

Paying attention to the health and well-being of employees in workplaces has been advocated in multiple occupations for numerous years. Studies that focused on nurses investigated this topic as a singular concept and have not developed a conceptual validation model [6,7,8]. Hence, the aim of the present study was to investigate the health and perception of work environment satisfaction amongst nurses as follows:

- To investigate the relationship between well-being and work environment satisfaction;
- To investigate the relationship between health-promoting lifestyles and work environment satisfaction; and
- To investigate the relationship between health-promoting lifestyles and well-being.

The results of this study will provide an understanding to develop a basis for proposing strategies in nursing administration management.

### 1.1. Correlation Between Well-Being and Work Environment Satisfaction

Well-being refers to a person’s perceptions of influential factors in the surroundings. A recent study proposed equilibrium theory, which posits that people’s well-being increases (decreases) if they can (cannot) use their resources to balance the challenges they encounter [9]. Well-being, a multidimensional concept comprising various factors, is a subjective experience to describe satisfaction in life.

Various measurement instruments are used for evaluating work environment satisfaction across multiple workplaces in different professions. Begat et al. [10] employed several major dimensions to survey well-being in order to examine nurses’ satisfaction with their work environments; the results revealed that nurses’ well-being was positively correlated with their work environments. A positive nursing work environment must incorporate intervention measures that can effectively alleviate job stress, fatigue, as well as enhance nurses’ well-being [11,12]. Among the aforementioned studies, some have proposed that satisfaction within the work environment is positively associated with well-being. However, the applicability of cultural and professional attributes in validating this concept has yet to be established. The implication of nurses’ well-being also has not been well-defined. Presently, the methods for measuring the concept of nurses’ well-being still require further development and rigorous validation. Thus, the following hypothesis was proposed in this study:

**Hypothesis** **1.** **(H1).** *Nurses’ work environment satisfaction is positively correlated with their sense of well-being.*

### 1.2. Correlation Between Health-Promoting Lifestyles and Work Environment Satisfaction

Recent studies on public health indicate that lifestyle exerts substantial positive health effects. Therefore, the most essential health promotion strategy is to assist people to develop healthy lifestyles. A study conducted in the United Kingdom revealed that 69.1% of nurses were obese (95% confidence interval (CI): 64.6, 73.6), a percentage considerably higher than that for nonmedical professions (68.9%; CI: 68.1, 69.7). Obesity influences nurses’ health and their roles as professionals in health promotion [13].

Nurses’ lifestyles are associated with unhealthy behaviors, and specific examples of such behaviors are a lack of exercise and unhealthy diets, which lead to inadequate physical fitness. Failure to address this problem may lead to health problems in nurses, consequently influencing the quality of health care they provide. Although nurses are crucial assets to hospitals and are frontline care providers for patients, little attention is devoted to their personal health. Moreover, direct evidence for validating the association between health-promoting lifestyles and satisfaction within the work environment remains scant. Additionally, the correlation between nurses’ health-promoting lifestyles and satisfaction within the work environment must be validated. Therefore, the following hypothesis was proposed in this study:

**Hypothesis** **2.** **(H2).** *Nurses’ health-promoting lifestyles and their work environment satisfaction are positively correlated.*

### 1.3. Correlation Between Health-Promoting Lifestyles and Well-Being

According to the theory of the health promotion model proposed by Pender et al. [14], a health-promoting lifestyle is a type of approach behavior. Specifically, it refers to the behaviors adopted by people to maintain or increase their well-being and achieve self-realization and personal accomplishments. Interpersonal relationships act to enhance people’s psychological well-being during work as an effect of the health-promoting lifestyle. Assisting people to develop positive coping skills and improved psychological health is crucial, as it is a valuable resource for enhancing people’s subjective well-being [15]. As such, Brunges et al. and Kuoppala et al. [16,17] have suggested that health promotion programs in workplaces should incorporate strategies that improve employees’ physical and psychosocial health. Measurable constructs have yet to be described that accurately correlate health-promoting lifestyles and well-being for nurses. Furthermore, the variables specific to nurses’ job characteristics and well-being have yet to be established for validating the correlation. As such, the following hypothesis was proposed:

**Hypothesis** **3.** **(H3).** *Nurses’ health-promoting lifestyles and their sense of well-being are positively associated.*

**Figure 1:**
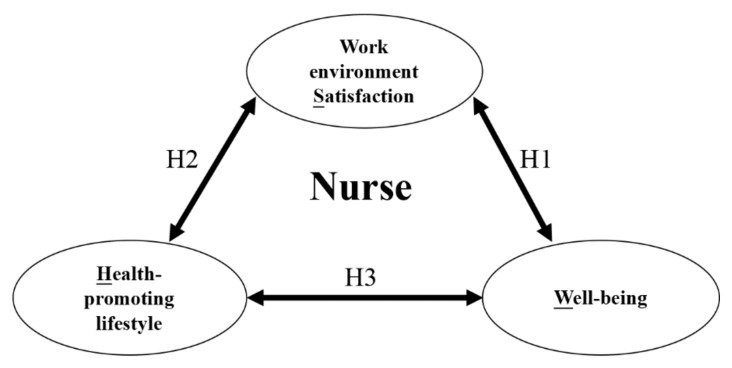
Conceptual Framework Illustrating the Relationships among Nurses’ Well-being, Health-promoting lifestyles, and Work Environment Satisfaction.

Based on previous research and the proposed hypotheses, this study submits the following framework (Figure 1) to validate the established measurement and structural models: Nurses’ well-being, health-promoting lifestyles, and work environment satisfaction (WHS; Figure 1).

## 2. Materials and Methods

### 2.1. Design and Participants

A cross-sectional correlational survey was conducted to develop measurement scales and the structural model. Sample size was decided according to the minimum requirement of sample sizes of 100, 200, and 200 for exploratory factor analysis (EFA), confirmatory factor analysis (CFA) and structural equation modeling (SEM), respectively, proposed by Thompson [18]. A total sample size of 750 was estimated for collecting the 500 (i.e., 100+200+200) valid questionnaires by considering a 67% return rate. Registered nurses in a Taiwanese medical center were recruited via stratified random sampling, with departments as strata. Through comprehensive participant–researcher communication, the study purpose, procedures, and time schedule for the questionnaire administration were explained to the nurses. The nurses who were willing to attend the study came to the arranged meeting room according to the arranged time schedule. After they signed the informed consent form, the study researcher distributed them the questionnaire. The participants were also asked to fill their personal information including age, marital status, education level, and tenure. During the questionnaire completion, the participants were allowed to discontinue at any time if they had doubts or felt uncomfortable. They subsequently returned the filled questionnaires to the researcher after they completed the questionnaire. The researcher then checked and validated if the information filled was complete and reliable. Subsequently, 750 self-administered questionnaires were distributed to the nurses, and finally a total of 672 valid questionnaires (return rate: 89.6%) were obtained. The study was approved by the Research Ethic Committee of Buddhist Tzu Chi General Hospital with the consent form number of IRB099-68.

### 2.2. Measures and Variables

After consulting previous studies [10,14,15], the present study designed a new scale in Chinese to assess nurses’ experience (Nursing Health and Job Satisfaction (NHJS) scale) for assessing hospital nurses’ well-being, health-promoting lifestyles, and satisfaction work environment satisfaction. Five experts assessed the content validity of the self-administered questionnaire (content validity index = 0.85). The items and the corresponding constructs of the final validated version of the scales are summarized in Table S1 (Supplementary Material). The scales are explained briefly as follows.

- Well-being subscale. This Likert scale comprised six items and two constructs (contentment and joyfulness), with the items scored from 5 (strongly agree) to 1 (strongly disagree). The range of the scores was 6–30, with a higher score representing a higher level of well-being.
- Work environment satisfaction subscale. This Likert scale involved five constructs (benefit, respect, reward, support, and guarantee) measured using 16 items, with the items scored from 5 (strongly agree) to 1 (strongly disagree). The range of the scores was 16–80, with a higher score representing higher satisfaction.
- Health-promoting lifestyle subscale. This Likert scale involved three constructs (physical activity, positive value, and willingness to share) measured using nine items, with the items scored from 5 (strongly agree) to 1 (strongly disagree). The range of the scores was 9–45, with a higher score representing a greater tendency toward leading health-promoting lifestyles.

### 2.3. Data Analysis

The IBM SPSS Statistics for Windows (Version 19.0. IBM Corp: Armonk, NY, USA) and AMOS for Windows (Version 19.0. IBM Corp: Armonk, NY, USA) were employed to analyze the data. The independent samples *t*-test was performed to test differences between each item’s means of the upper 27% scores group and lower 27% scores group defined as participants with the top 27% and bottom 27% of total scores of the scale, respectively, in order to evaluate item discrimination ability. EFA was performed to extract the constructs of the scales. CFA was performed for validating the reliability and validity of the scales obtained from the EFA. To validate the conceptual model in Figure 1, SEM was adopted to estimate the path coefficients of the exogenous and endogenous latent variables of the structural model and to validate the goodness of fit and strength of association of the model, The goodness-of-fit index (GFI), χ2/df, the comparative fit index (CFI), and root mean square error of approximation (RMSEA) served as the basis for examining the goodness of fit of the proposed model [19]. A *p* value < 0.05 was considered statistically significant.

## 3. Results

### 3.1. Demographics of the Sampled Participants

Of the distributed questionnaires, 672 valid responses were obtained, yielding a return rate of 89.6%. The respondents who provided valid responses were randomly divided into two groups—(1) the reliability and validity of the scales were analyzed for group one (*N* = 376, with EFA conducted on 176 respondents and CFA conducted on 200 respondents), and (2) the WHS conceptual framework was validated through SEM in group two (N = 296). As indicated in Table 1, the demographic characteristics among the respondents subjected to EFA, CFA, and SEM analyses were similar. Among the 672 respondents, 451 (67.1%) were aged 30 years or younger, 204 (30.4%) were married, 406 (60.4%) held a bachelor’s degree or higher, and 205 (30.5%) had two or fewer years of work experience.

### 3.2. Reliability and Validity of the Scales

A summary of the EFA and CFA results are presented in Table 2. Before the implementation of EFA, the upper 27% and lower 27% score groups for each item were compared. The results for the statistically significant difference for all items showed good item discrimination ability for all items. In EFA, the orthogonal rotation with varimax method was employed to extract the structure for each of three scales. Through EFA, the number of items for each of the three subscales was reduced from 24 to 18 (well-being), 40 to 33 (satisfaction within work environment), and 38 to 26 (health-promoting lifestyle). Simultaneously, constructs of two, five, and four were extracted, respectively. The Kaiser–Meyer–Olkin (KMO) values were 0.89–0.95, indicating that factor analysis was suitable for the content of all three subscales. The percentage of total variance explained ranged from 92.6% to 95.1%, which suggests good ability of the scales to explain the variability in responses after reduction and extraction through EFA. CFA was conducted for validating the structure of scales extracted from EFA. For each of three scales, the items with factor loading less than 0.5 were deleted due to lack of convergent validity. The item “I arrange suitable vacations and travel activities” with factor loading of 0.49 in the health-promoting lifestyle scale was not deleted since its factor loading was very close to 0.5. After CFA, the items/variables were further reduced from 18 to six (well-being), 33 to 16 (satisfaction within work environment), and 26 to nine (health-promoting lifestyle scale). The well-being subscale incorporates two constructs (joyfulness and contentment), the work environment satisfaction subscale incorporates five constructs (benefits, support, respect, security, and facilities), and the health promoting lifestyle subscale incorporates three constructs (activities, attitude, and companionship). Among the three scales, the Cronbach α values ranged from 0.83 to 0.91, indicating high internal consistency. The composite reliability was in the range 0.57–0.89, the standardized factor loading ranged from 0.49–0.95, and the average variance extracted was 0.54–0.89, signifying that the latent variables explained a high percentage of the variance in the observed variables, suggesting acceptable convergent validity. Discriminant validity ranged from 0.76 to 0.89, signifying that each scale construct was distinctive. All model fit indices (χ2/df = 1.60–1.99, RMSEA = 0.045–0.089, GFI = 0.94–0.98, and CFI = 0.96–0.98) demonstrated favorable goodness of fit of the measurement model (see Table 2). The measurement results by CFA for each item of the NHJS scale can be seen in Table S2 (Supplementary Material).

### 3.3. Findings Regarding Hypothetical Model

**Figure 2:**
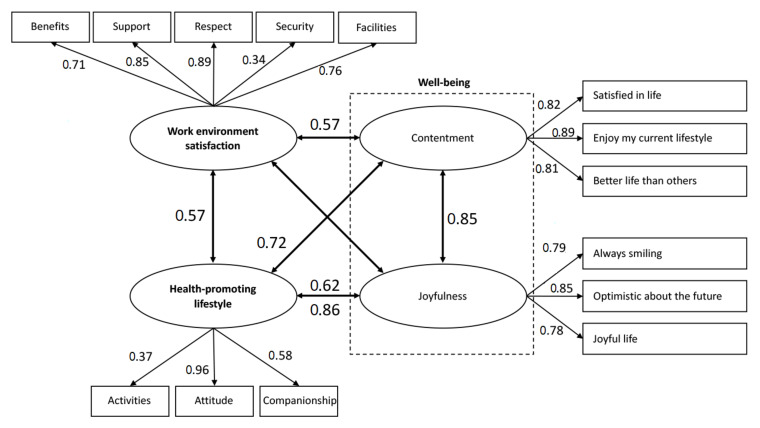
Hypothesized structural model with the obtained path analysis results.

The conceptual model with the obtained path analysis results are provided in Figure 2. The model fit assessment results (χ2/df = 1.71, RMSEA = 0.05, GFI = 0.89, and CFI = 0.95) indicated that the hypothesized structural model fitted the data.

The results of path analysis (Table 3) supported H1, H2, and H3 in terms of the relationships among well-being, nurses’ work environment satisfaction, and health-promoting lifestyles. The coefficients obtained from the structural model indicated that satisfaction within the work environment was positively and significantly correlated with the well-being constructs, contentment (CC = 0.57) and joyfulness (CC = 0.62). Work environment satisfaction was positively and significantly correlated with the nurses’ health-promoting lifestyle (CC = 0.57). Nurses’ health-promoting lifestyle was positively and significantly correlated with the two well-being constructs, contentment (CC = 0.72) and joyfulness (CC = 0.86). Furthermore, contentment and joyfulness were positively and significantly correlated (CC = 0.85) within well-being, as shown in Figure 2.

Regarding the strength of the correlation coefficient of each construct, health-promoting lifestyles exhibited stronger correlations with the two well-being constructs, compared with work environment satisfaction (CC = 0.72/0.86 vs. 0.57/0.62, see Figure 2). The moderate positive correlation (CC = 0.57) between health-promoting lifestyles and work environment satisfaction indicated the positive impact of health-promoting lifestyles on the work environment satisfaction. Exploring the three constructs of health-promoting lifestyles revealed that attitude exhibited the highest explained variation (standardized regression weight = 0.96). Among the five constructs of satisfaction within the work environment, respect and support demonstrated the highest explained variation (standardized regression weights = 0.89 and 0.85, respectively).

## 4. Discussion

The objective of this study was to develop and validate the WHS conceptual model for enhancing nurses’ health and well-being in a work environment with high work stress. During the study, the NHJS scale (Chinese version) for evaluating hospital nurses’ well-being, health-promoting lifestyles, and work environment satisfaction was developed and validated. The scale may serve as a tool to assess the psychological health status and potential for nurse attrition by hospital human resource management. Among research programs on nursing administration in clinical practice, this paper is the first to present a conceptual model and validated scale to assess hospital nurses and the relationship among health-promoting lifestyle, well-being and work environment satisfaction.

The health-promoting scale proposed by [14] involves six constructs (spiritual growth, exercise, health responsibility, nutrition, interpersonal support, and stress management), and by contrast the NHJSS simplified the constructs into three main categories—(1) activities, (2) attitude, and (3) companionship. Nurses’ health and retention in hospital wards remains critical in order to offer quality professional medical aid to patients. The study proposed a subscale (work environment satisfaction subscale) of the NHJS scale, comprising five constructs and 16 items, to measure nurses’ work environment satisfaction. Diverse factors are associated with satisfaction within the work environment, and the NHJS scale is able to accurately assess these factors. Moreover, the validation results with high percentages of the total variance explained in 92.6%–95.1% showed that the three subscales have excellent ability for evaluating the levels of well-being, work environment satisfaction, and health-promoting lifestyle. Reliability and validation analyses revealed that the NHJS scale is viable. Therefore, the NHJS scale can serve as an effective and useful instrument in nursing management and administration.

Among the three constructs of health-promoting lifestyles, attitude exhibited the higher explained variation. This construct includes the following items—“I am enthusiastic and optimistic about life”, “I feel that I am developing myself in a positive direction”, and “I feel self-confident”. When nurses are active, positive, and confident, their sense of well-being increases, thus increasing their satisfaction within the work environment and intent to remain at the hospitals. Meanwhile, among the five constructs of satisfaction within work environment, respect and support demonstrated the highest explained variation. These two constructs comprised the following items—“my job is respected by health professionals in the division”, “division supervisors offer assistance and are concerned on my behalf”, and “my job is respected by the supervisors of the division” for respect; “the institution supports nurses’ participation in academic conferences”, “the counseling resources is satisfactory when I conduct academic studies”, and “I receive adequate and professional assistance when I encounter difficulties at work” for support. The results reveal that nursing supervisors’ concern and respect, and institution’s support for work and academic growing for nurses are the main critical factors influencing nurses’ work environment satisfaction.

Enhancement of employees’ work motivation and organizational performance can be realized only when balance is maintained between employees’ personal needs and actual work demands [20,21]. Nursing is integral in promoting the well-being of patients. Currently, new-generation nurses hold different perceptions toward pursuing their own health and quality of life. By contrast, the previous commonly held notion was that nursing employees should be over-committed to their jobs. Therefore, in order to reduce the rate of attrition amongst nursing personnel, a healthy balance between work and lifestyle cannot be neglected. Institutions each have their own missions and values, both of which are crucial factors for employee retention. The current study proved the significant importance of nurses’ health-promoting lifestyle to their work environment satisfaction. The derived constructs in the WHS model indicated nurses’ active, positive, confident attitude plays a key role in promoting a healthy lifestyle. Furthermore, their work environment satisfaction increases if they get enough respect from other medical staff in work place. When nurses are satisfied with work environments, they are willing to dedicate themselves to achieve institutional missions and live up to institutional values. The institutional demands regarding the professional performance of employees do not necessarily exert negative stress on the employees. Instead, they may act to motivate them as well as enhance their job satisfaction and willingness to continue employment [22].

The main strength of the study is that this is the first study to present a conceptual model and validated scale to assess hospital nurses and the relationship among health-promoting lifestyles, well-being and work environment satisfaction. The study provides important insight which can be helpful for nursing administrators to improve nurses’ lifestyles. The developed NHJS scale also may facilitate the assessment of nurses’ psychological health status and potential for nurse attrition by hospital human resource management.

Several study limitations existed to reduce the applicable scope of this research. The main limitation is that only one hospital was available to recruit participants due to financial limitations. Although favorable internal validity was obtained based on one hospital, the external validity was unfavorable. A cross-sectional study design was not possible and therefore continuous longitudinal research data cannot be offered. As the NHJS scale is currently only available in Chinese, in its validated form, it is not available for further use or testing outside of Asia. To improve the relevance of the scale and items, it would be useful to incorporate variables such as nurses’ willingness to stay in their jobs or reasons nurses choose to quit.

## 5. Conclusions

Through the development of the NHJS scale, the validated WHS model demonstrates the positive correlation among hospital nurses’ health-promoting lifestyle, well-being, and work environment satisfaction. When nurses’ well-being is enhanced, their work environment satisfaction also increases. Human resources encourage nurses’ health-promoting lifestyles, as it positively benefits their well-being and satisfaction within the work environment. Nurses’ attitude plays a key role in promoting a healthy lifestyle. The work environment satisfaction variable that proves most important for improved sense of well-being and possible reduction in attrition rates is respect from other medical staff. These findings and the NHJS scale can serve as an empirical basis and measurement instrument for hospital nursing administrators. The NHJS scale makes it possible for hospital administrators to accurately assess and improve their decision-making process to aid in reducing nurse attrition rates and enhancement of nurses’ health.

## Figures and Tables

**Table 1:** Demographic Characteristics of the Questionnaire Respondents.

Variables	Total *n* = 672		EFA *n* = 176		CFA *n* = 200		SEM *n* = 296	
	*n*	%	*n*	%	*n*	%	*n*	%
Age								
<30 y/rs	451	67.1	121	68.8	133	66.5	197	66.6
≥30 y/rs	221	32.9	55	31.3	67	33.5	99	33.4
Marital status								
Married	204	30.4	51	29.0	67	33.5	86	29.1
Others	468	69.6	125	71.0	133	66.5	210	70.9
Education								
College	266	39.4	76	43.2	79	39.5	111	37.5
Bachelor or above	406	60.4	100	56.8	121	60.5	185	62.5
Tenure								
≤2 y/rs	205	30.5	66	37.5	52	26.0	87	29.4
> 2 y/rs	467	69.5	110	62.5	148	74.0	209	70.6

EFA: Exploratory factor analysis, CFA: Confirmatory factor analysis, SEM: structural equation modeling.

**Table 2:** EFA and CFA Results for the NHJS Scale.

Variables		1. Well-Being	2. Work Environment Satisfaction	3. Health-Promoting Lifestyle
EFA				
KMO ^1^ value		0.95	0.94	0.89
Constructs/items		2/18	5/33	4/26
Percentage of TVE^2^ explained		92.6%	95.1%	94.0%
CFA				
Constructs/items		2/6	5/16	3/9
Internal consistency	Cronbach’s α	0.91	0.91	0.83
Reliability	CR ^3^ > 0.6	0.85~0.86	0.78~0.89	0.57~0.83
Convergent validity	FL ^4^ > 0.5	0.78~0.89	0.70~0.92	0.49~0.95
	AVE ^5^ > 0.5	0.65~0.69	0.54~0.72	0.80~0.89
Discriminant validity	$\sqrt{\mathrm{AVE}}$ > *CC* ^6^	0.80~0.83	0.79~0.89	0.76~0.85
Model fit index	*X*^2^/df: 1–5	1.99	1.6	3.5
	GFI ^7^ > 0.9	0.98	0.94	0.96
	CFI ^8^ > 0.9	0.98	0.98	0.96
	REMSEA ^9^ < 0.1	0.089	0.045	0.092

^1^ KMO: Kaiser–Meyer–Olkin, ^2^ TVE: Total variances explained, ^3^ CR: composite reliability, ^4^ FL: factor loading, ^5^ AVE: average variance extracted, ^6^ CC: correlation coefficient, ^7^ GFI: Goodness-of-fit index. ^8^ CFI: Comparative fit index, ^9^ RMSEA: Root mean square error of approximation (RMSEA).

**Table 3:** Correlation Coefficient of the Structural Relationships for the WHS Model.

Hypothesis		CC	S.E.	Z	*p*-Value
H1	Satisfaction within work environment and joyfulness are positively correlated.	0.62	0.03	6.76	<0.001 ***
	Satisfaction within work environment and contentment are positively correlated.	0.57	0.03	6.59	<0.001 ***
H2	Satisfaction within work environment and health promotion lifestyle are positively correlated.	0.57	0.02	5.50	<0.001 ***
H3	Health promotion lifestyle and joyfulness are positively correlated.	0.86	0.04	6.74	<0.001 ***
	Health promotion lifestyle and contentment are positively correlated.	0.72	0.04	6.49	<0.001 ***

CC: correlation coefficient, S.E.: standard error, ***: *p*-value < 0.01

## Supplementary Materials

The following are available online at https://www.mdpi.com/1660-4601/17/10/3582/s1, Table S1. Nursing Health and Job Satisfaction (NHJS) Scale; Table S2. Measurement Results for the Nursing Health and Job Satisfaction (NHJS) Scale.
