# Supplementary material for: Nurses’ Well-Being, Health-Promoting Lifestyle and Work Environment Satisfaction Correlation: A Psychometric Study for Development of Nursing Health and Job Satisfaction Model and Scale
Source: Int J Environ Res Public Health. 2020 May 20;17(10):3582. doi: 10.3390/ijerph17103582 (PMC7277543; doi:10.3390/ijerph17103582)
Supplement: Supplementary file 1 [file ijerph-17-03582-s001.pdf]

Table S1. Nursing Health and Job Satisfaction (NHJS) Scale

Subscale	Construct	Item
Well-being	Contentment	I am currently satisfied with everything in my life.
		I enjoy my current lifestyle.
		I often feel that I am leading a better life than others.
	Joyfulness	I am always smiling.
		I am optimistic about the future.
		I have lived a joyful life.
Work environment Satisfaction	Benefits	Benefits package is acceptable.
		Night-shifts salary is reasonable.
		The salary I receive is sufficient.
		Paid holiday time is sufficient.
	Support	The institution supports nurses' participation in academic conferences.
		The counseling resources is satisfactory when I conduct academic studies.
		I receive adequate and professional assistance when I encounter difficulties at work.
	Respect	My job is respected by health professionals in the division.
		Division supervisors offer concern and assistance to me.
		My job is respected by the supervisors of the division.
	Security	The accident reporting and prevention system in the institution is adequate.
		Methods employed by the institution for managing dangerous items are safe and suitable.
		Regulations stipulated by the institution for managing sexual harassment incidents are efficient.
	Facilities	Efficiency of the institution in supplying consumable medical materials.
		Sufficient medical equipment provided by the institution.
		Essential safety equipment for infection control provided by the institution.
Health-promoting lifestyle	Activities	I perform stretching exercises at least three times per week.
		I perform strenuous exercises at least three times per week.
		I engage in leisure physical activities.
	Attitude	I am enthusiastic and optimistic about life.
		I feel that I am developing myself in a positive direction.
		I fell self-confident.
	Companionship	I am happy to maintain contact with those I am close to.
		I arrange suitable vacations and travel activities.
		I keep in contact with my family and/or love relationships.

Table S2. Measurement results for the Nursing Health and Job Satisfaction (NHJS) scale

Subscale	Construct	Item	Unstandardized estimate	Standard error	Standardized estimate	*p* value
Well-being CR = 0.926 AVE = 0.679 α = 0.91	Contentment α = 0.872	I am currently satisfied with everything in my life.	1	~	0.82	
		I enjoy my current lifestyle.	1.27	0.07	0.89	<0.001 ***
		I often feel that I am leading a better life than others.	1.12	0.07	0.81	<0.001 ***
	Joyfulness α = 0.846	I am always smiling.	1.07	0.08	0.79	<0.001 ***
		I am optimistic about the future.	1.17	0.08	0.85	<0.001 ***
		I have lived a joyful life.	1	~	0.78	
Work environment satisfaction CR = 0.951 AVE = 0.665 α = 0.91	Benefits α = 0.889	Benefits package is acceptable.	1	~	0.92	
		Night-shifts salary is reasonable.	0.89	0.05	0.71	<0.001 ***
		The salary I receive is sufficient.	1.02	0.04	0.91	<0.001 ***
		Paid holiday time is sufficient.	0.81	0.06	0.70	<0.001 ***
	Support α = 0.837	Efficiency of the institution in supplying consumable medical materials.	1	~	0.72	
		Sufficient medical equipment provided by the institution.	1.04	0.08	0.83	<0.001 ***
		Essential safety equipment for infection control provided by the institution.	1.08	0.08	0.85	<0.001 ***
	Respect α = 0.839	My job is respected by health professionals in the division.	1	~	0.80	
		Division supervisors offer concern and assistance to me.	0.99	0.07	0.71	<0.001 ***
		My job is respected by the supervisors of the division.	1.10	0.08	0.82	<0.001 ***
	Security α = 0.868	The accident reporting and prevention system in the institution is adequate.	1.11	0.07	0.90	<0.001 ***
		Methods employed by the institution for managing dangerous items are safe and suitable.	0.82	0.06	0.74	<0.001 ***
		Regulations stipulated by the institution for managing sexual harassment incidents are efficient.	1	~	0.85	
	Facilities α = 0.883	Efficiency of the institution in supplying consumable medical materials.	1	~	0.80	
		Items and numbers of medical equipment provided by the institution.	1.04	0.06	0.89	<0.001 ***
		Essential safety equipment for infection control provided by the institution.	1.10	0.07	0.86	<0.001 ***
Health-promoting lifestyle CR = 0.940 AVE = 0.643 α = 0.83	Activities α = 0.799	I perform stretching exercises at least three times per week.	1.04	0.05	0.73	<0.001 ***
		I perform strenuous exercises at least three times per week.	0.96	~	0.75	
		I engage in leisure physical activities.	1.00	0.05	0.80	<0.001 ***
	Attitude α = 0.886	I am enthusiastic and optimistic about life.	1.01	~	0.87	
		I feel that I am developing myself in a positive direction.	1.00	0.07	0.91	<0.001 ***
		I fell self-confident.	0.87	0.06	0.78	<0.001 ***
	Companionship α = 0.785	I am happy to maintain contact with those I am close to.	1.00	~	0.95	
		I arrange suitable vacations and travel activities.	0.58	0.10	0.49	<0.001 ***
		I keep in contact with my family and/or love relationships.	0.95	0.12	0.85	<0.001 ***

***: p-value < 0.01.
